# CIB2 mediates acquired gefitinib resistance by inducing ZEB1 expression and epithelial-mesenchymal transition

**DOI:** 10.18632/aging.206086

**Published:** 2024-09-10

**Authors:** Feng-Mei Zhou, Kun-Kun Wang, Li-Hong Wang, Jian-Ge Qiu, Wei Wang, Wen-Jing Liu, Lin Wang, Bing-Hua Jiang

**Affiliations:** 1 Academy of Medical Science, Zhengzhou University, Zhengzhou 450000, China; 2 The Affiliated Cancer Hospital of Zhengzhou University, Zhengzhou 450000, China

**Keywords:** non-small cell lung cancer, gefitinib, CIB2, EMT, tumor growth

## Abstract

EGFR-TKIs have been used as frontline treatment in patients with advanced non-small cell lung cancer (NSCLC) suffering from the *EGFR* mutation. Gefitinib, the first-generation EGFR-TKI, has greatly improved survival rates in lung cancer patients, whereas acquired gefitinib resistance is still a critical issue that needs to be overcome. In our research, high expression levels of CIB2 were found in gefitinib-resistant lung cancer cells. CIB2 knockout rendered gefitinib-resistant cells more sensitive to gefitinib, and overexpression of CIB2 in parental cells was sufficient to induce more resistance to gefitinib. Inhibition of CIB2 in gefitinib-resistant lung cancer cells significantly induced cell apoptosis. To clarify the major molecular mechanism by which CIB2 increases gefitinib resistance, we demonstrated that raised CIB2 in lung cancer cells promoted epithelial-to-mesenchymal transition (EMT) through upregulation of ZEB1. Moreover, FOSL1 transcriptionally regulated CIB2 expression. Finally, CIB2 rendered tumors resistant to gefitinib treatment *in vivo*. Our results explored a new mechanism: upregulated CIB2 promoted EMT through ZEB1 to regulate gefitinib resistance, which could be a candidate therapeutic target for overcoming acquired resistance to EGFR-TKIs in NSCLC patients.

## INTRODUCTION

Lung cancer is one of the most morbid and deadly cancers in the world, which induces a great threat to human health and life [1]. Approximately 80% of all lung cancers are non-small cell lung cancer (NSCLC) [2]. However, about 75% of NSCLC patients miss the optimal time to operate and are diagnosed at an advanced stage when they are first diagnosed [3]. For advanced lung cancer, targeted therapy has been a promising strategy [4, 5]. Epidermal growth factor receptor (EGFR) is a critical therapeutic target used to treat lung cancer [6, 7]. With the development of molecular biology and gene diagnosis, it has been found that there are mutations in EGFR in most patients with lung cancer [8], so basic medical and clinical studies have been carried out to target EGFR mutations [5, 9]. Gefitinib is a novel targeted therapy for the treatment of advanced or metastatic NSCLC, particularly for patients with mutations in EGFR exon 19 and/or exon 21 [10–12]. Although TKIs have brought new vitality to patients with lung cancer and improved the survival rate of patients, patients may develop acquired drug resistance to gefitinib after 6–12 months, which greatly reduces the survival rate of patients [12, 13]. Therefore, it is particularly important to understand the mechanism of gefitinib resistance in lung cancer and to find effective strategies for reversing drug resistance.

Calcium and integrin binding protein 2 (CIB2) is a small molecular EF-Hand protein that can bind to magnesium and calcium ions. It is widely expressed in multiple tissues, conveying that it may be directly involved in several physiological processes and diseases [14]. CIB2 has been reported to regulate Ca2+ homeostasis in sensory neurons of the ear and eye [15]. Additionally, other diseases and processes, including congenital muscular dystrophy type 1A3, have been linked to altered CIB2 function [16]. To elucidate the possible novel mechanisms of gefitinib resistance in lung cancer, we performed RNA sequencing on parent cells (PC-9) and gefitinib-resistant lung cancer cells (PC-9G). Compared to sensitive PC-9 cell lines, CIB2 expression levels were found to be significantly increased in PC-9G. However, its biological function in cancer development and chemoresistance is undetermined.

The aim of this study is to investigate the role of CIB2 in lung cancer and chemoresistance. The following questions will be discussed: (1) whether CIB2 is essential for lung cancer growth and gefitinib resistance; (2) to investigate whether CIB2 regulates ZEB1 expression to induce gefitinib resistance; (3) to verify whether the aberrant expression of CIB2 is regulated by transcription factor FOSL1 and the role and mechanism of FOSL1/CIB2/ZEB1 pathway in lung cancer development and resistance to gefitinib. Thus, our study will identify potential biomarkers and provide a theoretical basis for diagnosing lung cancer at an early stage and overcoming acquired resistance to gefitinib.

## MATERIALS AND METHODS

### Cell culture and tissue samples

PC-9 lung cancer cells were purchased from ATCC and cultured in an incubator containing 5% CO_2,_ which were grown in RPMI 1640 medium at 37°C. To obtain gefitinib-resistant PC-9G cells, PC-9 cells were cultured with different concentrations of gefitinib for six months. For knockout or overexpression of CIB2, stable cell lines were transfected using CIB2 sgRNA or CIB2 cDNA construct respectively, then selected by treating the cells with 10 μg/ml puromycin for 2 weeks. We obtained the lung cancer tissues and adjacent normal tissues from the Biobank of the Affiliated Cancer Hospital of Zhengzhou University (Zhengzhou, China). The tumor tissues were collected during the last several years, coded using bar codes and stored in the Biobank. The patients with these types of cancer did not receive any surgical treatment, radiotherapy, chemotherapy or immunotherapy prior to surgery.

### Cell proliferation assay

The cell proliferation rate was analyzed using CCK-8 solution kit (Vazyme Biotech, Nanjing, China), 10 μL of which was added to each well at 0, 24, 48 and 72 hours and incubated for 2 to 4 hours at 37°C. Absorbance was measured with a microplate reader at 450 nm.

### Transwell assay

In the migration assays, aliquots of suspended 5 × 10^4^ cells were cultured in the upper chamber of a Transwell (Corning, NY, USA) containing serum-free medium (*n* = 3). To test the cell invasion activity, Transwell chambers were coated with Matrigel for 30 minutes (BD Biosciences, CA, USA), then aliquots of the cells (1 × 10^5^) were seeded with serum-free medium. The lower chambers were filled with the complete medium. Upper chambers were removed after 16 to 18 hours of incubation, migrating/invading cells were fixed with 4% polyoxymethylene, imaged and calculated in randomly selected fields for further analysis.

### Cell apoptosis assay

For apoptosis assay, cells were seeded in 6-well plates, and treated with appropriate concentrations of gefitinib for 48 hours. Cells and culture medium were collected in tubes and centrifuged for 5 min at 1500 rpm. The cells were then washed twice with cold PBS and stained with Apoptosis Detection Kit. Cell apoptosis rates were analyzed with flow cytometry at room temperature within one hour.

### Plasmid construction

The CIB2 overexpression plasmid was constructed by cloning into the pCDH-EGFP vector. However, CIB2 knockout vector was constructed by CRISPR/Cas9 system. The lentiCRISPR/V2 vector was digested with BsmBI in 55°C NEBuffer for 2 hours, and the oligos of CIB2 were annealed according to the manufacturer’s instruction (NEB, MA, USA). The annealed product and the digested product of lentiCRISPR/V2 were linked in 1 × T4 DNA ligase Buffer (NEB, B0202A) with T4 DNA ligase overnight at 16°C. The plasmids were isolated according to the manufacturer’s instruction (QIAGEN, Hilden, Germany).

### Luciferase reporter assay

To determine the transcriptional activity of FOSL1 to affect CIB2 expression, CIB2-WT and CIB2-MUT-sequences were inserted into pGL3 plasmid. The FOSL1 overexpression plasmid was co-transfected into HEK293T with CIB2-WT or CIB2-MUT reporter plasmids. The cells were incubated for 48 hours, and the luciferase activity was assayed using a dual luciferase assaying system kit according to the manufacturers’ guidelines.

### Western blotting

Cells/tissues were lysed using RIPA lysis buffer and the protein was collected by centrifuging at 13,000 rpm for 15 min at 4°C. After estimating protein concentration by BCA assay kit (Thermo Ficher Scientific, Waltham, MA, USA), total protein samples were separated by 10% SDS-PAGE and transferred to PVDF membranes (Millipore Corporation, Bedford, MA, USA). Membranes were incubated with appropriate specific primary antibodies overnight at 4°C after blocking with 5% BSA for 1 hour at room temperature. Secondary antibodies were then added to the membranes. Finally, an Imaging System was used to visualize the band signals.

### Tumor xenograft assay

For xenograft experiments, we used 4-week-old female BALB/c nude mice dividing into four groups and subcutaneously injected 5 × 10^6^ cells resuspending in 100 μL serum-free medium to the flanks of the mice (*n* = 4). We began to evaluate the tumor volumes every two days and the volume of the tumor was calculated using the formula (width2 × length)/2. Finally, we harvested the xenograft tumor after three weeks. All animal care and methods were in accordance with institutional guidelines and were approved by the Animal Care and Use Committee of Zhengzhou University.

### Statistical analysis

GraphPad Prism 8 was used to analyze the data in this study and expressed as mean ± SD of three replicates. Furthermore, the Student’s *t*-test was used for the analysis of quantitative variables between the two groups, with differences considered significant at *P* < 0.05.

## RESULTS

### CIB2 expression was dramatically increased in gefitinib-resistant cells and higher CIB2 levels in lung cancer patients were linked to poor prognosis

**Figure 1:**
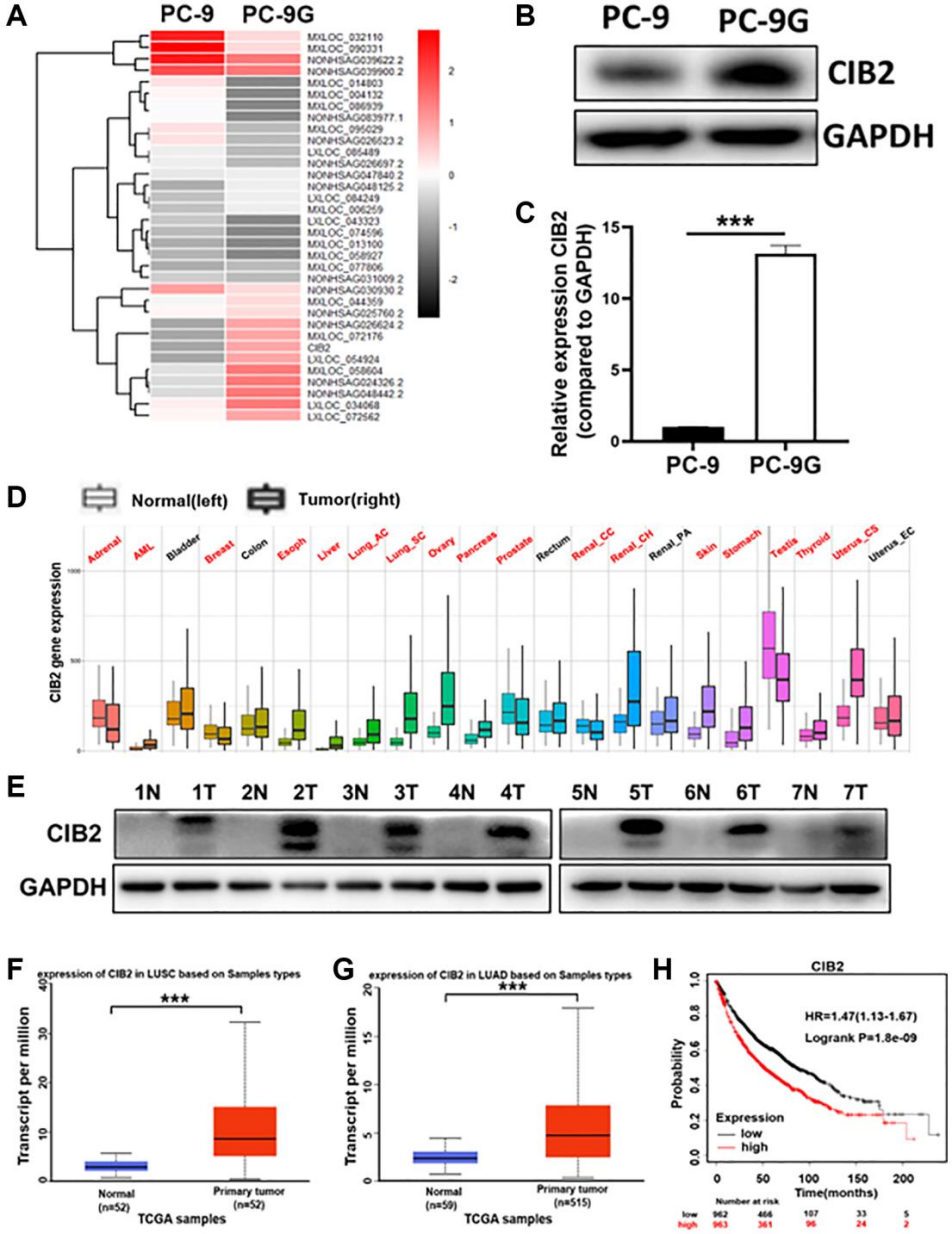
**CIB2 expression was dramatically increased in gefitinib-resistant cells and higher CIB2 levels in lung cancer patients were linked to poor prognosis.** (**A**) Heat map of RNA-seq results (PC-9G vs. PC-9). (**B**, **C**) CIB2 mRNA and protein expression levels were detected by Western blotting and qRT-PCR in PC-9 and PC-9G cells. (**D**) The CIB2 expression levels were analyzed and compared in tumor and normal tissues of 22 human cancers by pan-cancer analysis through TNM plot (https://tnmplot.com/analysis/). Significant differences by Mann-Whitney *U*-test are marked with red. (**E**) The protein expression of CIB2 in normal and tumor samples obtained from the Biobank. GAPDH levels were used as an internal loading control. (**F**, **G**) CIB2 expression in patients of LUAD (normal = 52, tumor = 52) and LUSC (normal = 59, tumor =515) were examined in TCGA database. (**H**) Kaplan-Meier plot of the overall survival of patients with lung cancer with high or low expression of CIB2 from Kaplan-Meier plotter (http://kmplot.com/analysis/). Data were statistically analyzed using Student’s *t*-test and values were presented as mean ± SD. ^***^indicated significant difference at *p* < 0.001.

To clarify the possible new mechanism of acquired gefitinib resistance, RNA sequencing was performed with gefitinib-sensitive parent cells (PC-9) and gefitinib-resistant PC-9G cells, and the result suggested that CIB2 expression was obviously upregulated in PC-9G (Figure 1A). We further showed that CIB2 protein expression levels were dramatically increased in PC-9G cells compared to PC-9 (Figure 1B). The mRNA levels of CIB2 were also obviously increased in PC-9G compared to the parental cells (Figure 1C). To figure out the function of CIB2 in tumor progression, CIB2 levels were analyzed in multiple cancers, and CIB2 levels were up-regulated in pan-cancer samples compared to controls (Figure 1D). Similarly, CIB2 was highly expressed in lung cancer samples when compared to normal samples (Figure 1E). In addition, UALCAN database also conveyed that the CIB2 levels were greatly increased both in lung adenocarcinoma (LUAD) and lung squamous carcinoma tissues (LUSC) (Figure 1F, 1G). The correlations of the patient survival and CIB2 levels in lung cancer tissues were analyzed using The Kaplan-Meier plotter database, and higher CIB2 levels highly correlated with lower overall survival rates (Figure 1H), and higher CIB2 levels indicated poor overall survival rates in the LUAD patients in the TCGA database (Supplementary Figure 1). CIB2 was also obviously increased in the gefitinib-resistant cells than sensitive cells in GSE169513 (Supplementary Figure 2). Thus, our results conveyed that the expression levels of CIB2 were induced in gefitinib-resistant cells, which could be a potential biomarker to predicting gefitinib resistance in the future.

### CIB2 increases gefitinib resistance by inhibiting cellular apoptosis

**Figure 2:**
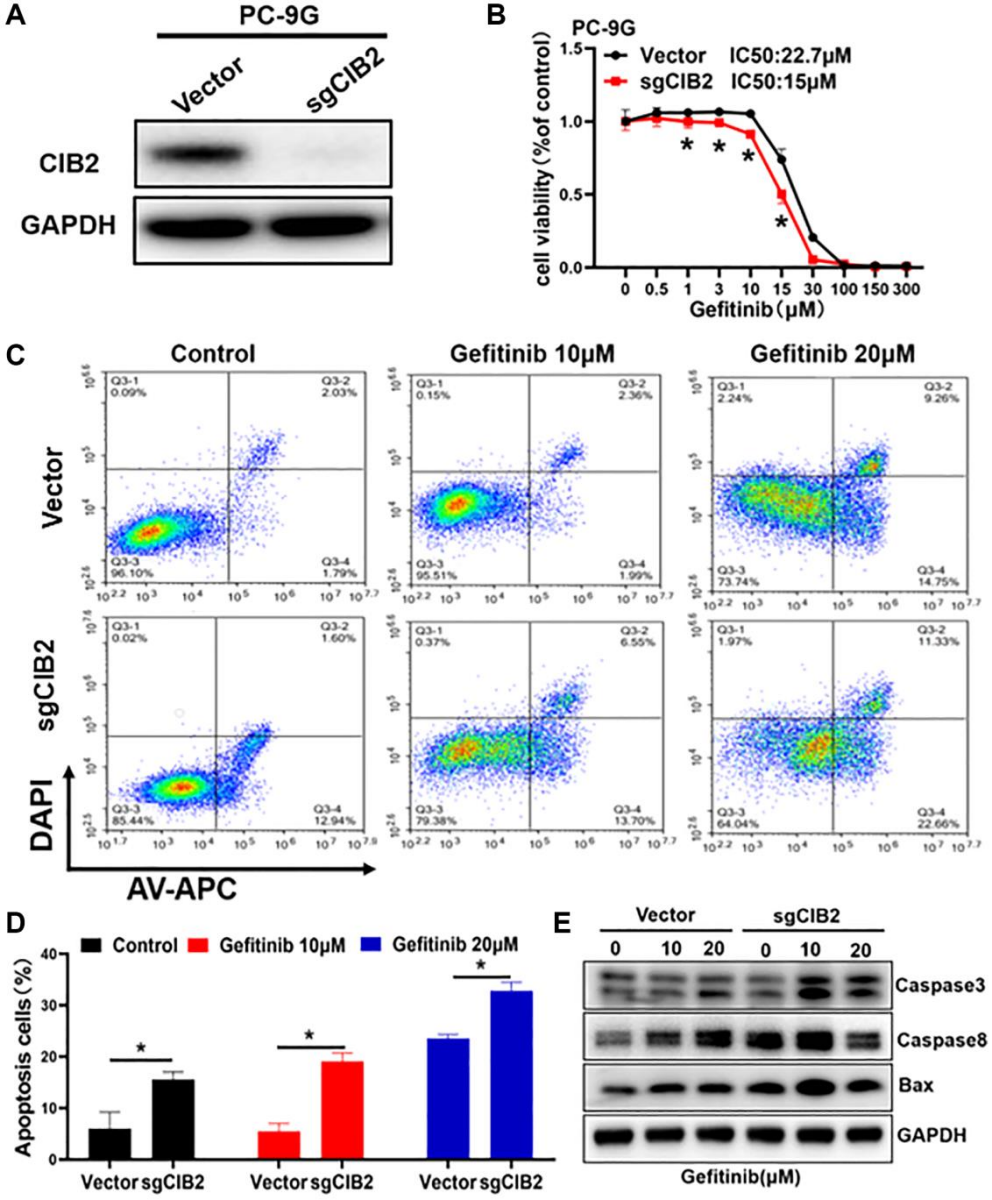
**CIB2 increases gefitinib resistance by inhibiting cellular apoptosis.** (**A**) CIB2 knockout stable cell lines were constructed by lentivirus infection, and the expression levels of CIB2 were analyzed by Western blotting. (**B**) The gefitinib sensitivities were analyzed in CIB2 knockout cells treated with different concentrations of gefitinib for 72 h using CCK8 kit. (**C**) CIB2 knockout cells were treated with different gefitinib or PBS for 48 h, then the proportion of cell apoptosis were measured by flow cytometry. (**D**) Cell apoptosis rates were quantitatively calculated by histogram. (**E**) The protein expression levels of BAX, Caspase3 and Caspase8 were detected by Western blotting in cells with CIB2 knockout. Data were statistically analyzed using Student’s *t*-test and values were presented as mean ± SD of three independent experiments. ^*^indicates significant difference at *p* < 0.05.

To demonstrate the role of CIB2 in gefitinib resistance, we established CIB2 knockout cell lines (Figure 2A), then cells were treated with gefitinib at different concentrations for 72 h. The result showed that CIB2 knockout increased the gefitinib sensibility in PC-9G cells (Figure 2B). On the contrary, force expression of CIB2 in PC-9 cells induced gefitinib resistance (Supplementary Figure 3A, 3B). These results showed that CIB2 is important for inducing gefitinib resistance. We then explored the potential mechanism of CIB2, and the cells were treated using different concentrations of gefitinib in CIB2 knockout and control groups, the rates of apoptotic cells from the CIB2 knockout group were significantly increased compared to control (Figure 2C, 2D). To study the mechanism of CIB2 regulating cell apoptosis, we further examined the Caspase3, Caspase8 and BAX protein levels by Western blotting analysis, and CIB2 knockout significantly upregulated the levels of Caspase3, Caspase8 and BAX (Figure 2E). In conclusion, these data suggested that CIB2 may function in promoting gefitinib resistance by inhibiting cell apoptosis.

### CIB2 acted as an oncogene in lung cancer development

**Figure 3:**
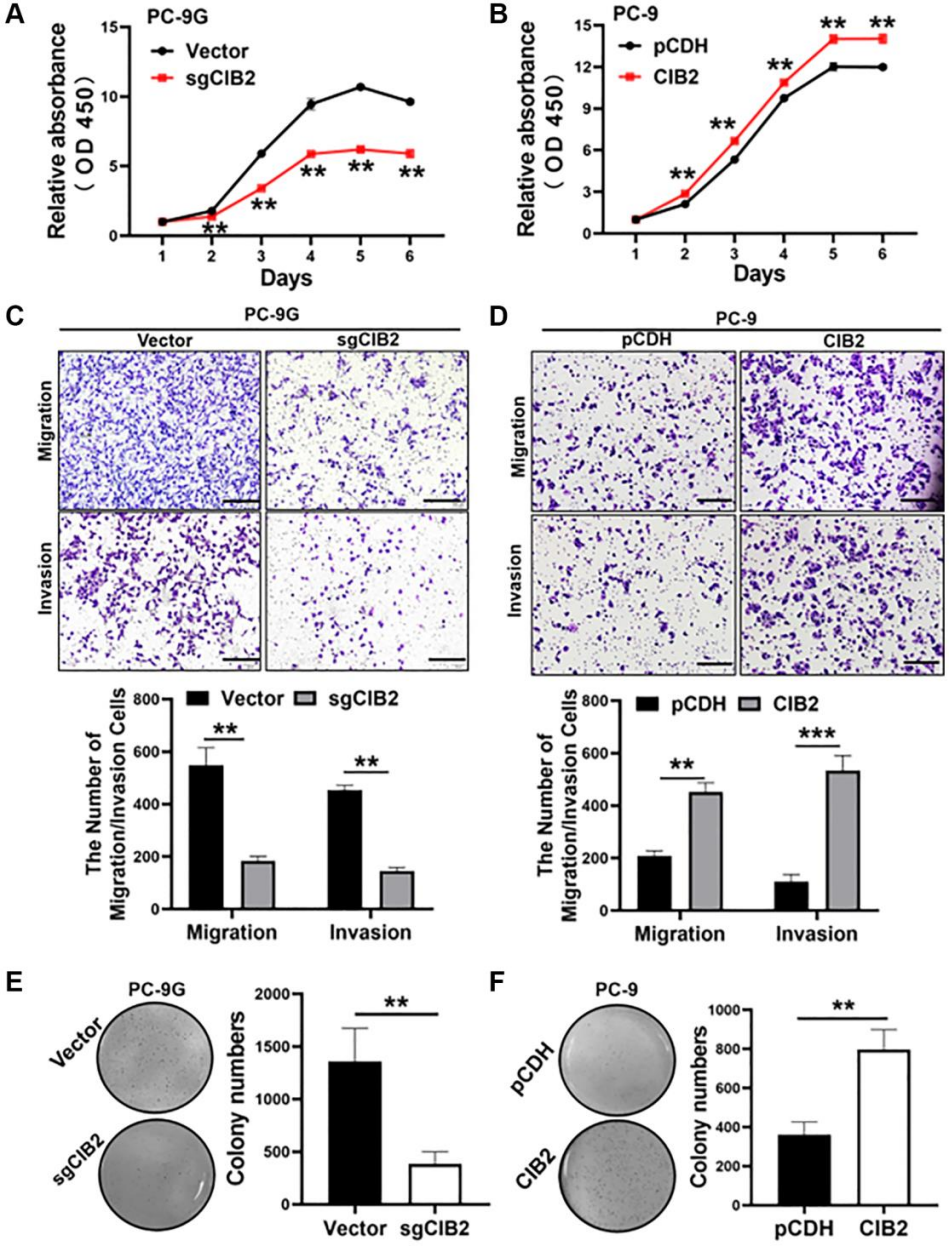
**CIB2 acted as an oncogene in lung cancer development.** (**A**, **B**) Cell proliferation abilities of CIB2 knockout or overexpression were measured using the CCK8 assay. (**C**, **D**) Cell migration and invasion activities of indicated cells were analyzed using the Transwell assay. (**E**, **F**) Indicated cells were used to test colony formation activities with soft agar assay. Data were statistically analyzed using Student’s *t*-test and values were shown as mean ± SD of three independent experiments. ^**^indicated significant difference at *p* < 0.01, ^***^indicated significant difference at *p* < 0.001.

To investigate the function of CIB2 in lung cancer cells, we first detected that CIB2 knockout PC-9G cells grew more slowly and CIB2 overexpression in the PC-9 cells promoted cell proliferation (Figure 3A, 3B). To figure out whether CIB2 regulated cell migration and invasion, cells were cultured in the Transwell chamber, and the results suggested that CIB2 knockout in PC-9G cells dramatically suppressed cell migration and invasion activities (Figure 3C). Besides, CIB2 overexpression in the cells promoted cell migration and invasion activities (Figure 3D). Also, the results showed that CIB2 knockout obviously inhibited the abilities of PC-9G cells to form colonies through colony formation assays (Figure 3E), and CIB2 overexpression increased colony formation activities (Figure 3F). Thus, our findings conveyed that CIB2 functioned as an oncogene in lung cancer and promoted tumor development.

### CIB2 increases the expression levels of ZEB1, EMT-associated marker to promote gefitinib resistance

**Figure 4:**
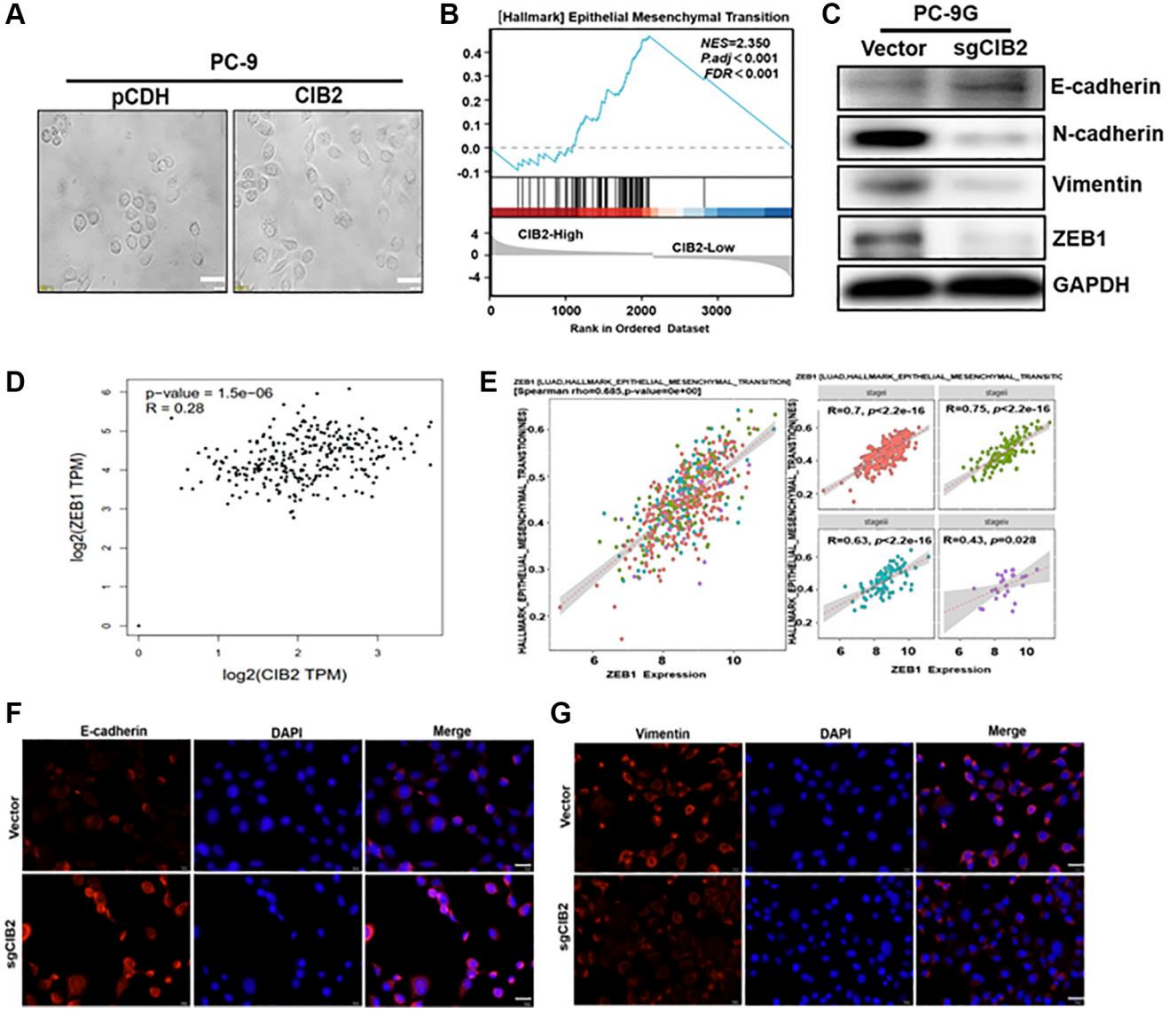
**CIB2 increases the expression levels of ZEB1, EMT-associated marker to promote gefitinib resistance.** (**A**) Morphological changes were tracked by microscopy in CIB2-overexpressed cells. Scale bar = 100 μm (×400 magnification). (**B**) GSEA program was used to analyze HALLMARK_Epithelial–Mesenchymal Transition pathway enrichment scores between CIB2 high- and low-expression groups using TCGA lung cancer dataset. (**C**) The expression of N-Cadherin, Vimentin, E-Cadherin, and ZEB1 was determined using Western blotting. (**D**) Correlation analysis of the TCGA dataset to assess expression of CIB2 and ZEB1 in lung cancer tissue using the GEPIA database (http://gepia.cancer-pku.cn/index.html). (**E**) The correlation analysis of the ZEB1 expression and HALLMARK_Epithelial Mesenchymal Transition pathway in lung cancer tissues (https://bio.tools/emtome). (**F**, **G**) Immunofluorescence assay (IF) was used to detect E-cadherin and Vimentin levels in CIB2 knockout stable cells compared to control cells.

More interestingly, force expression of CIB2 rendered the changes in cell morphology were obviously detected in PC-9 cells, and upregulating CIB2 showed a spindle shape compared to control (Figure 4A), which accounting for that CIB2 may play a role in regulation of EMT pathway. GSEA analysis showed that higher CIB2 levels were corelated with EMT pathway in lung cancer tumor tissues in the cohort (Figure 4B). EMT can be recognized by changes in numerous critical molecular markers, particularly the depletion of E-cadherin and the acquisition of N-cadherin or Vimentin. To verify whether CIB2 influenced the EMT process, we first examined the expression levels of the EMT-related markers E-cadherin, N-cadherin and Vimentin in PC-9 and PC-9G cells. Indeed, Western blotting analysis showed that ZEB1, Vimentin and N-cadherin levels were increased in PC-9G cells, while E-cadherin levels were obviously decreased in PC-9G cells (Supplementary Figure 4A). We further detected that ZEB1, Vimentin and N-cadherin were greatly decreased, with E-cadherin expression upregulated in CIB2 knockout cells compared to the control (Figure 4C). In CIB2 overexpression cells, ZEB1, Vimentin and N-cadherin levels were greatly increased, as well as E-cadherin level was significantly decreased in CIB2-overexpressed cells (Supplementary Figure 4B). In addition, the positive correlation of CIB2 and ZEB1 in lung cancer samples was analyzed with the GEPIA database (Figure 4D). ZEB1 expression levels have been shown to be correlated with EMT pathway activation in lung cancer tissues (Figure 4E), suggesting the crucial role of CIB2/ZEB1 in tumor metastasis. Further study by immunofluorescence staining also showed that CIB2 knockout decreased Vimentin expression and increased E-cadherin level (Figure 4F, 4G). These results suggest that CIB2 induced EMT procession through regulating ZEB1 expression, suggesting the importance of CIB2/ZEB1 regulatory axis in lung cancer development and gefitinib resistance.

### ZEB1 functioned as a downstream regulator in CIB2-induced EMT and chemoresistance

**Figure 5:**
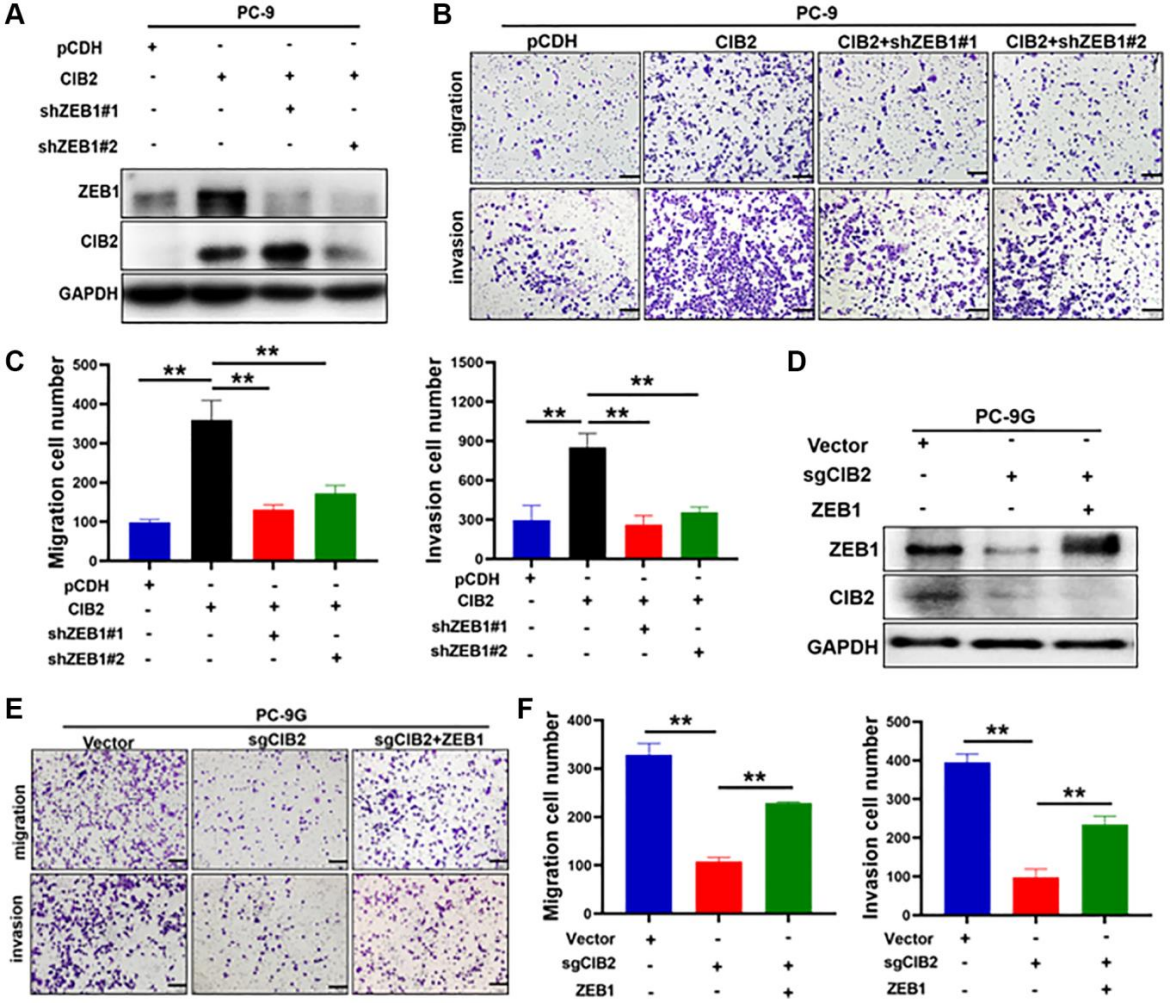
**ZEB1 functioned as a downstream regulator in CIB2-induced EMT and chemoresistance.** (**A**) PC-9 cells with stably overexpressed CIB2 were transfected with shZEB1#1 or shZEB1#2 for 48 hours. Western blotting was used to detect the protein expression levels of ZEB1 and CIB2. (**B**, **C**) Cell migration and invasion abilities were measured after silencing ZEB1 in CIB2-overexpressed stable PC-9 cells. Quantitative analysis of migration and invasion cell numbers were shown in histogram. (**D**–**F**) PC-9G cells with CIB2 knockout were transfected with ZEB1 overexpression plasmid. Cell migration and invasion abilities were measured and analyzed. Data were statistically analyzed using Student’s *t*-test and values were shown as mean ± SD of three independent experiments. ^**^indicated significant difference at *p* < 0.01.

EMT has been reported to function in cell migration, invasion, and drug resistance in several types of cancer. Numerous studies show that ZEB1 is crucial for regulating the EMT. To verify whether CIB2/ZEB1 regulated cell migration and invasion, we performed ZEB1 knockdown by transfecting ZEB1 shRNA into CIB2-overexprssed PC-9 cells (Figure 5A). Our results suggested that ZEB1 knockdown inhibited the migration and invasion abilities in PC-9 cells with CIB2 overexpression (Figure 5B, 5C). Further study showed that CIB2 knockout in PC-9G cells suppressed cell migration and invasion, and the inhibitory effect could be partly reversed by overexpression of ZEB1 (Figure 5D–5F). Thus, these data demonstrated the CIB2/ZEB1 axis functioned as a vital regulator in EMT and chemoresistance of lung cancer cells.

### FOSL1 increased CIB2 expression levels at the transcriptional level

**Figure 6:**
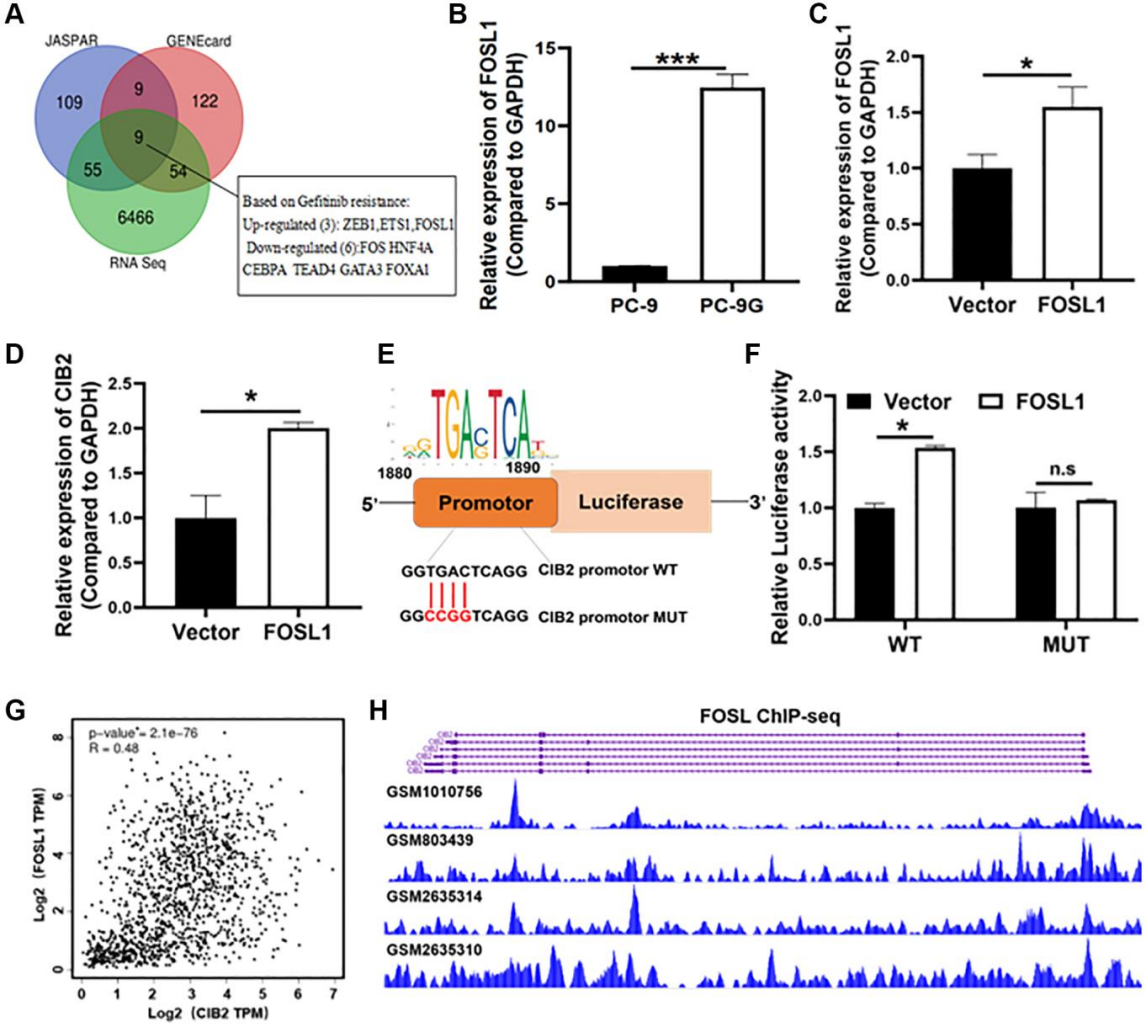
**FOSL1 increased CIB2 expression levels at the transcriptional level.** (**A**) Venn diagram showing overlap between RNA sequence data (PC-9 vs. PC-9G) and the CIB2 upstream transcription factor predicted by two websites: JASPAR (http://jaspar.genereg.net/) and Gene Cards (https://genecards.com/). (**B**) Relative mRNA expression levels of FOSL1 were detected by qRT-PCR in PC-9 and PC-9G cells. (**C**, **D**) The FOSL1 and CIB2 expression levels were detected in FOSL1-overexpressed cells by qRT-PCR in PC-9 cells. (**E**) The WT/MUT binding sites and FOSL1 motif in the promoter region of CIB2 were shown. (**F**) The luciferase reporter assays were used to test CIB2-WT or CIB2- MUT binding activities with FOSL1. (**G**) Correlation relationships between CIB2 and ZEB1 were analyzed in the cBioPortal database (https://www.cbioportal.org/). (**H**) Cistrome database was used to show the binding peaks of FOSL1 at CIB2 promoter region (http://cistrome.org/db/#/). Data were statistically analyzed using Student’s *t*-test and values were shown as mean ± SD of three independent experiments. ^*^indicated significant difference at *p* < 0.05, ^***^ indicated significant difference at *p* < 0.001.

To further explore the molecular mechanism of CIB2 upregulation, we initially used two publicly available prediction databases via JASPAR and Gene Cards websites to analyze candidate transcription factors which may bind to the promoter region of CIB2, and found 9 transcription factors as potential candidates that were also interacted with our RNA sequence data from PC-9 and PC-9G cells (Figure 6A). FOSL1 showed the highest binding score with the CIB2 promoter. To verify whether FOSL1 functioned in activating the CIB2 expression, we showed that FOSL1 mRNA expression levels in PC-9-G cells were upregulated by 3-fold compared to PC-9 (Figure 6B). We further constructed stable cells overexpressing FOSL1, and RT-qPCR assay confirmed the stable cell line with FOSL1 overexpression (Figure 6C). Force expression of FOSL1 significantly promoted CIB2 levels (Figure 6D). To further confirm the molecular mechanism of CIB2 upregulation by FOSL1, we used JASPAR to predicate binding sites on the promoter region of CIB2 (Figure 6E). To further explore the direct interaction between CIB2 and FOSL1, the luciferase reporter experiment confirmed that FOSL1 increased the luciferase activities of CIB2, indicating that FOSL1 regulates the CIB2 expression through the transcriptional activation (Figure 6F). Then, we applied Cistrome database to check CIB2 promoter region with FOSL1 binding peaks through ChIP-Seq analysis. The data conveyed that FOSL1 binding peaks were enriched in CIB2 transcription start site (TSS) (Figure 6G). Interestingly, FOSL1 levels were found to have a positive relationship with CIB2 levels by correlation coefficient analysis using the GEPIA database in lung cancer patient samples (Figure 6H). These findings suggest that the upregulation of CIB2 is regulated by FOSL1.

### CIB2 expression promotes tumorigenesis and gefitinib resistance *in vivo*

**Figure 7:**
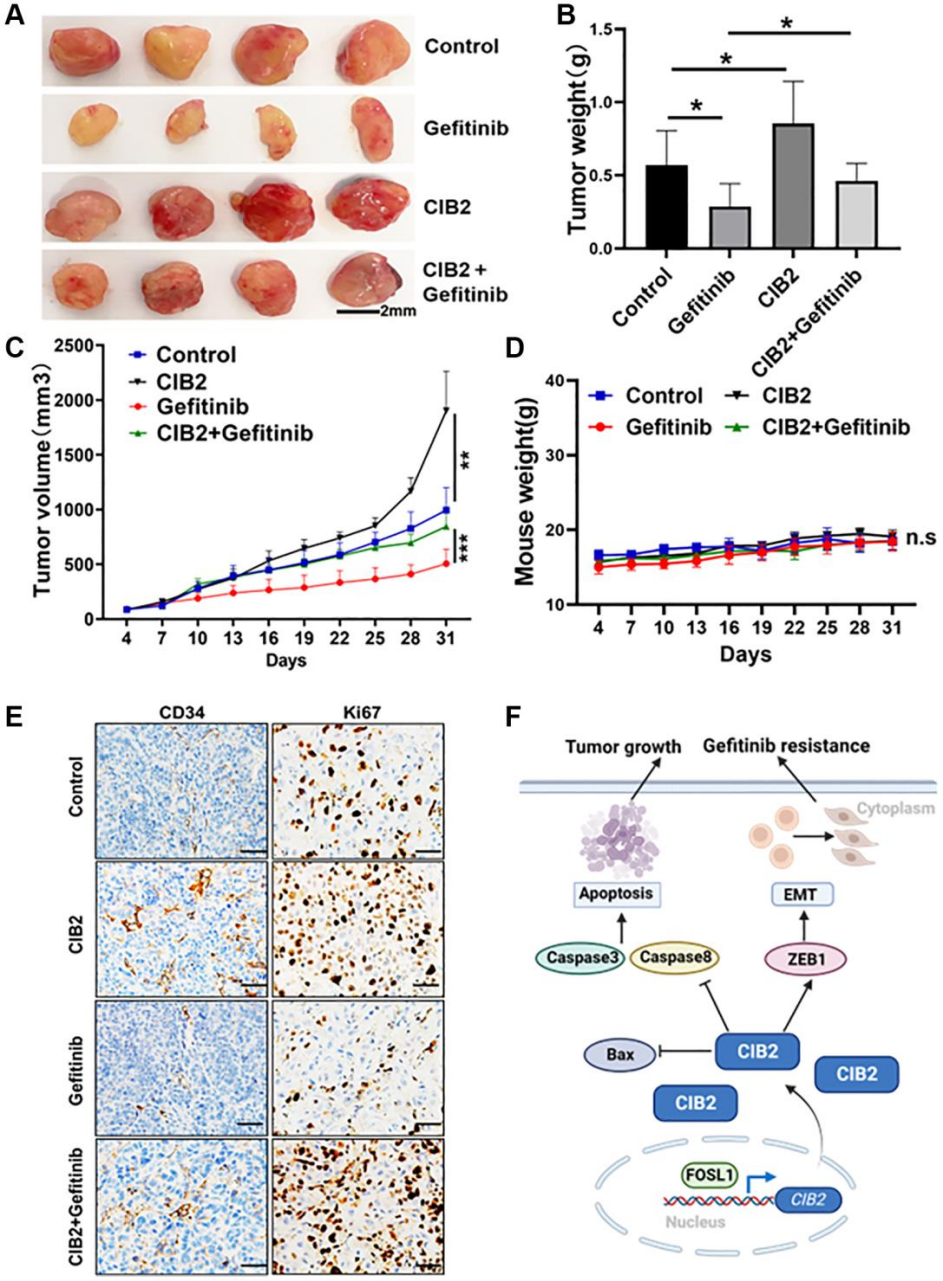
**CIB2 expression promotes tumorigenesis and gefitinib resistance *in vivo*.** PC-9 or CIB2-overexpressed cells were injected into the flanks of nude mice. When the tumor volume reached about 50 mm3, gefitinib (10 mg/kg) or 0.9% saline was given by oral gavage every three days. Mice weights and tumor volumes of mice were recorded. After three weeks, the mice were anaesthetized, and tumor tissues were stripped out and then weighted. (**A**) Each group of tumors was taken out for photography and analysis. Tumor weights (**B**) and tumor sizes (**C**) were significantly larger in CIB2-overexpressed group, despite given gefitinib to the CIB2 group, the tumor weights and tumor volumes showed resistance to gefitinib treatment. Corresponding control (*n* = 4). Data were statistically analyzed using Student’s *t*-test and values were presented as mean ± SD of three independent experiments. ^**^ and ^***^. ^*^indicated significant difference at *p* < 0.05. (**D**) Body weights of mice in each group showed no significant changes during the drug treatment. (**E**) The immunohistochemistry results showed high Ki67 and CD34 expression levels in CIB2-overexpressed group than the control group, and the CIB2-overexpressed group treated with gefitinib showed slightly decreased Ki67 and CD34 expression levels. (**F**) A pattern diagram to summarize the role of CIB2 in the regulation of tumorigenesis and chemoresistance in lung cancer. CIB2/ZEB1 axis induced tumor growth and gefitinib resistance by inhibiting cell apoptosis and enhancing EMT in lung cancer.

To study the role of CIB2 on tumorigenesis *in vivo*, we constructed a xenograft nude mice model with CIB2-overexpressed PC-9 cells and control cells. The four groups were treated with 10 mg/kg gefitinib or saline by gavage every three days. The tumor volumes were measured, and tumor weights were calculated at the end of the experiment; tumor weights were increased in CIB2-overexpressed group compared to control, and CIB2-overexpressed group treated with gefitinib showed a decline in tumor weights (Figure 7A, 7B). Our results further showed that the tumor volumes were larger in CIB2-overexpressed group compared to control, while treatment with gefitinib conveyed a slight growth inhibition in CIB2-overexpressed tumors compared to control (Figure 7C). Moreover, there were no obvious changes in mice weights (Figure 7D). Further study with IHC staining investigated that Ki-67 and CD34 levels were increased in the CIB2-overexpressed tumors compared with control, and CIB2-overexpressed tumors treated with gefitinib also showed a slight inhibition of Ki-67 and CD34 levels (Figure 7E). In Figure 7F, we draw a pattern diagram to summarize the regulatory mechanism of CIB2 in tumorigenesis and gefitinib resistance in lung cancer. CIB2 induced tumor growth and gefitinib resistance by inhibiting cell apoptosis and enhancing EMT in NSCLC, and CIB2 could be applied as a novel biomarker for diagnosis of drug resistance of lung cancer.

## DISCUSSION

EGFR-TKIs (including gefitinib) are frequently utilized in the clinical therapy of NSCLC. Although TKIs are initially effective, around 65% of EGFR-TKI-sensitive individuals with NSCLC develop resistance after 9–13 months following therapy [17, 18]. It is critical to improve the anticancer efficacy of targeted therapy for these individuals. It was discovered that, except for EGFR mutations that account for acquired EGFR-TKIs resistance in NSCLC, aberrant signal pathways or key oncogene-driven genes [19, 20], such as STAT3 activation [9], MET amplification [9, 21] and the transformation of phenotype, including EMT also linked to the TKIs drug resistance [9, 22, 23]. A recent work from our lab found that increasing NOX4 expression in gefitinib-resistant cells, and NOX4 reduction boosted the efficacy of gefitinib treatment [10]. Furthermore, earlier research found that an elevated level of STAT3 phosphorylation may trigger EGFR-TKI resistance in lung cancer patients [24, 25]. However, the biological processes behind acquired TKI resistance are unclear, and new key regulators or pathways responsible for EGFR-TKIs resistance remain to be explored.

In this research, we first suggested that CIB2 levels were increased in gefitinib-resistant cells. In ovarian cancer, CIB2 has been shown to operate as a possible tumor suppressor by decreasing tumor development and cell migration/invasion, therefore reduced CIB2 levels have been linked to a poor prognosis in patients [26]. Furthermore, CIB2 was found to play a role in negatively regulating Rheb-mTORC1 signaling axis, and subsequently essential to autophagy in age-related macular degeneration disease [27]. However, despite the high expression of CIB2 in gefitinib-resistant cells, the mechanism of CIB2 has not been revealed in lung cancer. Our research showed CIB2 knockout increased the sensitivity to gefitinib treatment, and upregulation of CIB2 decreased the sensitivity to gefitinib. Downregulation of CIB2 promoted cell apoptosis to enhance gefitinib sensitivity. Thus, our results first conveyed that CIB2 could be a key regulator in inducing gefitinib resistance, it is essential to explore the function and involved pathways regulated by CIB2 in gefitinib-resistant cells. Our investigation of CIB2 in cancer development and chemoresistance will provide a broad vision in lung cancer.

Our study also identified that FOSL1 is the upstream regulator of CIB2, a critical transcription factor which can directly bind to the promoter region of CIB2 to activate CIB2 expression. Therefore, whether CIB2 has other therapeutic implications in EGFR-TKIs resistance has not been confirmed, which needs further study. Cancer cells constantly communicate with their surroundings to avoid immune cell destruction which leads to treatment resistance and metastasis [28]. EMT has been linked to tumor invasion and metastasis [29, 30]. Several investigations have established that EMT causes resistance to chemotherapy or targeted therapy in cancer [31–33]. More interestingly, in our study, we observed that overexpressed CIB2 rendered cells in a spindle shape, which suggested that CIB2 influenced EMT to regulate gefitinib-resistance. Further study showed that ZEB1 was highly expressed in gefitinib-resistant cells. Upregulation of CIB2 induced ZEB1, Vimentin and N-cadherin levels, and reduced E-cadherin levels. Thus, CIB2/ZEB1 regulatory axis played a vital role in cell metastasis and drug resistance. In addition, the mRNA expression levels of CIB2 and ZEB1 were also upregulated in osimertinib-resistant lung cancer cell lines, which indicates that CIB2 and ZEB1 may contribute to osimertinib resistance.

In conclusion, our study first demonstrated that FOSL1/CIB2/ZEB1 pathway functioned in regulation of gefitinib resistance and CIB2 could be applied as a novel biomarker for diagnosis of drug resistance of lung cancer. Therefore, our study will provide a new strategy to overcome drug resistance and lung cancer tumorigenesis.

## Supplementary Materials

Supplementary Figures
