# Supplementary material for: CIB2 mediates acquired gefitinib resistance by inducing ZEB1 expression and epithelial-mesenchymal transition
Source: Aging (Albany NY). 2024 Sep 10;16(17):12277–92. doi: 10.18632/aging.206086 (PMC11424576; doi:10.18632/aging.206086)
Supplement: Supplementary Figures [file aging-16-206086-s001.pdf]

SUPPLEMENTARY FIGURES

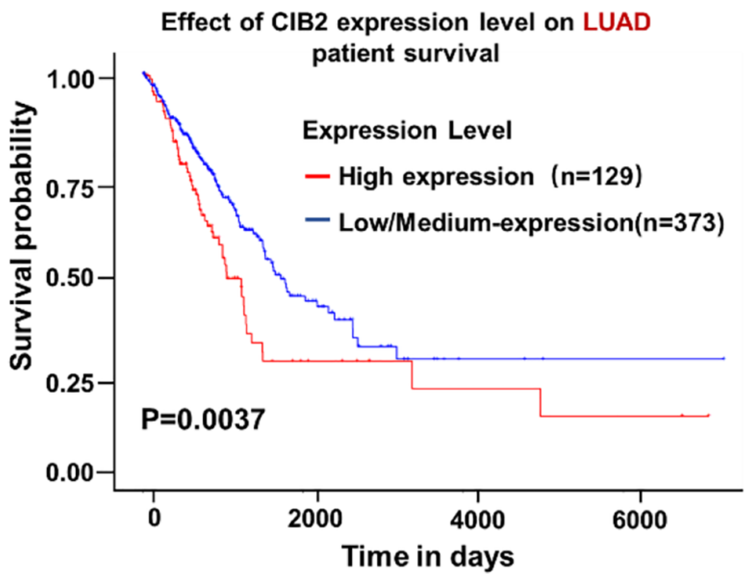

Supplementary Figure 1. Higher levels of CIB2 were significantly associated with the lower overall survival in the LUAD patients. The Kaplan–Meier curve analyses revealed that CIB2 mRNA levels were strongly associated with the overall survival, and higher levels of CIB2 were significantly associated with the lower overall survival rates in the LUAD patients in the TCGA database.

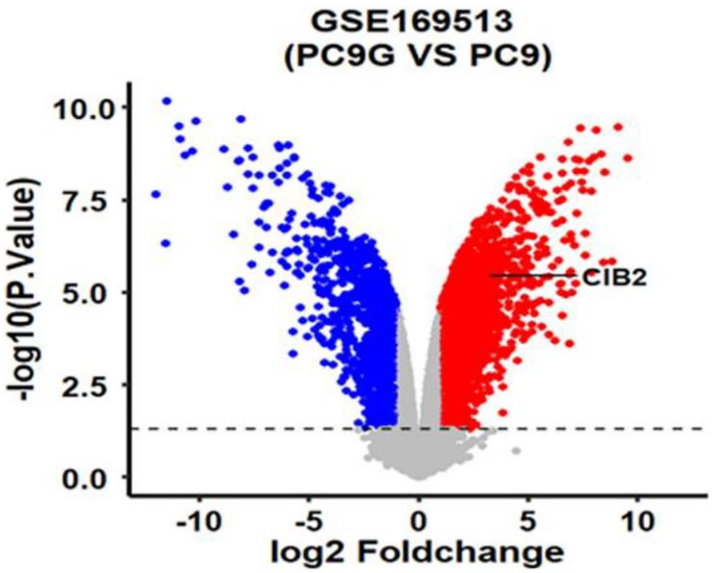

Supplementary Figure 2. CIB2 levels were significantly higher in gefitinib-resistant cells than sensitive cells. We analyzed the database from gene expression omnibus (GEO) and found that CIB2 levels were significantly higher in the gefitinib-resistant cells than those from gefitinib-sensitive cells (PC9G vs. PC9) in GSE169513.

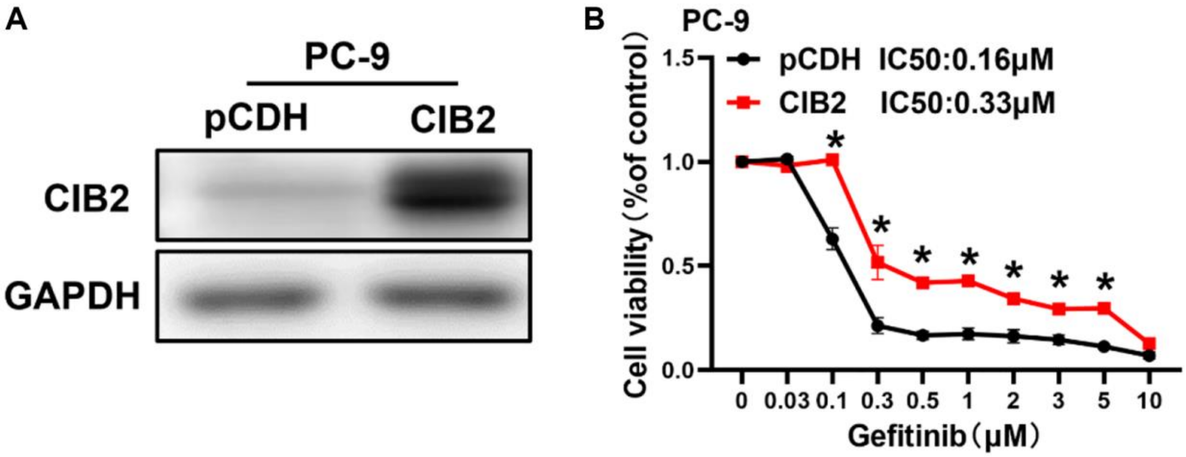

Supplementary Figure 3. Overexpression of CIB2 induced resistance to gefitinib treatment in PC-9 cells. (**A**) The CIB2 protein expression levels in stable CIB2-overexpressed PC-9 cells were analyzed by Western blotting. (**B**) The IC50 values of gefitinib sensitivity analysis in indicated cells were detected by CCK8. Data were statistically analyzed using Student's *t*-test and values were shown as mean ± SD. *indicates significant difference at $p < 0.05$.

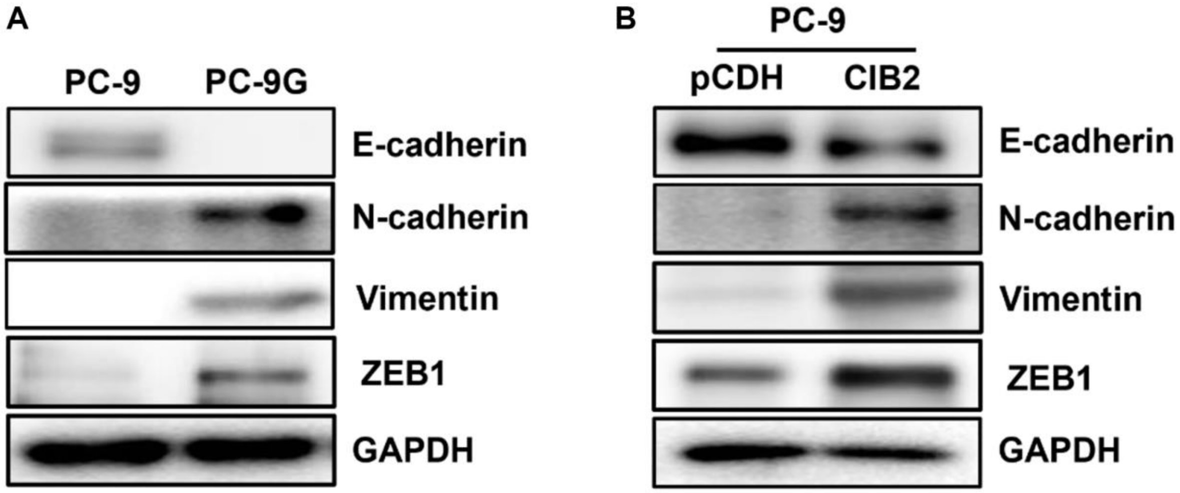

Supplementary Figure 4. Forced expression of CIB2 in parental cells induced Vimentin, N-cadherin and ZEB1 expression, but reduced E-cadherin levels. (**A**) The protein expression levels of E-cadherin, Vimentin, N-cadherin and ZEB1 in PC-9/PC-9G cells were analyzed by Western blotting. (**B**) The expression levels of EMT markers were shown in stable CIB2-overexpressed PC-9 cells compared to the vector control cells.
